# Profiling and factors associated with glaucoma diagnostic practice in sub-Saharan Africa-a cross sectional study of Nigerian and Ghanaian optometrists

**DOI:** 10.1186/s12886-023-03083-0

**Published:** 2023-08-08

**Authors:** Stephen Ocansey, Edgar Ekure, Uchechukwu L. Osuagwu, Bernadine N. Ekpenyong, Godwin Ovenseri-Ogbomo, Sylvester Kyeremeh, Kelechi C. Ogbuehi, Kingsley E. Agho, Khathutshelo P. Mashige, Antor O. Ndep, Kovin S. Naidoo, Centre for Eye Care, Public Health Intervention Initiative (CEPHII)

**Affiliations:** 1 Department of Optometry and Vision Science, School of Allied Health Sciences, College of Health and Allied Sciences, University of Cape Coast, Cape Coast, Ghana; 2 Department of Biomedicine, Salus University, 8360 Old York Road, Elkins Park, PA 19027 USA; 3 Bathurst Rural Clinical School (BRCS), School of Medicine, Western Sydney University, PO Box 9008, Bathurst, New South Wales 2795 Australia; 4 African Vision Research Institute, Discipline of Optometry, University of KwaZulu-Natal, Westville Campus, Durban, 3629 South Africa; 5 Epidemiology and Biostatistics Unit, Department of Public Health, University of Calabar, Calabar, Cross River State Nigeria; 6 Department of Optometry, Centre for Health Sciences, University of the Highlands and Islands, Inverness, IV2 3JH UK; 7 Department of Optometry and Visual Science, College of Science, KNUST, Kumasi, AK-385-1973 Ghana; 8 Department of Medicine, Dunedin School of Medicine, University of Otago, Otago, New Zealand; 9 School of Health Science, Western Sydney University, Campbelltown, NSW 2560 Australia; 10 Department of Public Health, Faculty of Allied Medical Sciences, College of Medical Sciences, University of Calabar, Calabar, Cross River State Nigeria; 11 School of Optometry and Vision Science, University of New South Wales, Sydney, NSW Australia

**Keywords:** Optometry, Glaucoma, Glaucoma assessment, Nigeria, Ghana

## Abstract

**Background:**

Ghana and Nigeria are the two countries in Africa that currently run the Doctor of Optometry (OD) program in sub-Saharan Africa (SSA). Optometrists in these countries are licensed to provide glaucoma care. Despite the clinically relevant practice guidelines for glaucoma, there is no data on the practice patterns for glaucoma eye care in SSA, a region with the highest prevalence of glaucoma. This study aimed to profile glaucoma diagnosis adherence to practice guidelines among optometrists in two neighbouring anglophone countries (Nigeria and Ghana).

**Methods:**

A web-based cross-sectional survey of practising optometrists was conducted in both countries. Each country data was weighted to reflect the total number of licensed and practising optometrists at the time of this survey. Descriptive analyses were performed against demography and practice factors using survey commands to adjust for sampling weights when estimating confidence intervals (CI) around prevalence estimates. Simple and multiple logistic regression analyses were performed to identify factors associated with glaucoma diagnosis.

**Results:**

A total of 493 optometrists (238, 48.3% and 255, 51.7%) from Ghana and Nigeria respectively, responded to the survey-the first to document and compare the glaucoma diagnostic criteria between optometrists in Ghana and Nigeria. More Ghanaian than Nigerian optometrists diagnosed glaucoma and over 90% in both countries reported that they frequently performed either tonometry, visual field testing, or fundus examination during glaucoma diagnosis. Ghanaian optometrists were significantly more likely to diagnose glaucoma than Nigerian optometrists (adjusted odd ratio, AOR = 6.15, 95%CI:1.63–23.15, *P* = .007). Optometrists who have practiced for more than 10 years (AOR = 7.04; 95%CI:1.74–28.47, *P* = .006) and private practice optometrists (AOR = 3.33; 95%CI:1.13–9.83, *P* = .03) were more likely to diagnose glaucoma.

**Conclusions:**

The study provides information for evaluating glaucoma assessment for optometrists in both countries. Optometrists in both countries are reasonably well-equipped to diagnose glaucoma and are practicing at an adequate level, but with room for improvement.

**Supplementary Information:**

The online version contains supplementary material available at 10.1186/s12886-023-03083-0.

## Background

Glaucoma is a multifactorial disease involving a group of progressive optic neuropathies characterized by apoptotic degeneration of retinal ganglion cell axons resulting in optic disc cupping, and a corresponding loss of vision [1]. Although the exact pathogenesis of the disease is still unknown the clinical features include generalized or focal loss of the neuroretinal rim tissues and thinning of the retinal nerve fiber layer resulting in characteristic visual field defect [2, 3]. This loss of the structural integrity of the optic nerve is associated with functional impairment, and if untreated, could lead to profound vision impairment and a consequent reduction in a patient’s quality of life [4].

Glaucoma is the second leading cause of irreversible blindness, accounting for about 8% of the 39 million blind individuals globally [5, 6]. The prevalence of glaucoma is highest in Africa at 4.4% compared to other parts of the world, and makes up about 15% of blindness in the region [6]. The high prevalence of visual impairment from glaucoma in Africa may be attributed to socio-economic factors, access to healthcare, awareness, early detection, and compliance to treatment [7, 8]. Furthermore, primary open angle glaucoma, the most prevalent form of glaucoma, much more insidious and usually asymptomatic until later in the disease process is reported to be associated with being of African descent [9, 10]. Differences in phenotypic characteristics together with larger disks, thinner CCTs and thinner RNFL have been suggested as reasons for the higher prevalence of POAG among Africans [11].

Early diagnosis and management of the glaucoma are essential to mitigating severe morbidity [12]. However, the complexity in diagnosing patients with early glaucoma is exacerbated by masquerade conditions such as arteritic and non-arteritic ischemic optic neuropathies, compressive optic nerve neuropathies, and hereditary optic neuropathies which share similar characteristics. A recent study found that 25% of non-glaucomatous optic neuropathies were misdiagnosed as glaucoma by glaucoma experts [13] suggesting that misdiagnosis may be higher among medical personnel with little training in glaucoma diagnosis. Rigorous evidence-based glaucoma practice is required to minimize the odds of misdiagnoses and improve early detection of glaucoma.

The American Academy of Ophthalmology, and American Optometry Association have developed evidence-based preferred practice patterns as a guideline for optimum care of glaucoma [14, 15]. United Kingdom [16], Australia, and New Zealand [17] have also developed similar standards of glaucoma care for ECPs. These recommended glaucoma diagnostic guidelines are reported in KF Jamous, M Kalloniatis, A Hayen, P Mitchell, FJ Stapleton and B Zangerl [18] paper.

A new multi-country practical toolkit for glaucoma management in sub-Saharan Africa (SSA) based on the International Council of Ophthalmology Glaucoma Guidelines, was developed by a consortium of experts from Africa [19]. This toolkit, is to be adopted by glaucoma care team members, including optometrists to set up integrated glaucoma care services adequate for their own context and to strengthen glaucoma care delivery in the region. Although periodic assessments of clinician adherence to these guidelines have been published for the afore-mentioned countries, no data exists for SSA, a region with a population of 1.1 billion people and the highest prevalence of primary open angle glaucoma globally.

This study was designed to evaluate the type of procedures used by Ghanaian and Nigerian optometrists in the diagnosis of glaucoma and whether they meet the preferred practice guidelines stipulated by the American Academy of Optometry and American Ophthalmology Association. The study also identified the factors affecting the diagnosis of glaucoma among respondents. Although Nigerian and Ghanaian optometrists are the only African countries with the highest level of optometric education in Africa and the optometrists in these countries practice at the World Council of Optometry competency level four [20] which includes provision of glaucoma care; they may not be deliberately managing glaucoma with the preferred patterns of practice in mind. This study will provide evidence on the level of glaucoma diagnostic practice among optometrists from the region to inform planning, areas of practice improvement and sustainability.

## Methods

The study adhered strictly to the principles of the 1967 Helsinki declaration (as modified in Fortaleza 2013) [21].

### Study area and setting

This study was conducted among practicing optometrists in the Western African countries of Nigeria and Ghana. In Nigeria, a country of about 206, 139, 587 people which shares borders with Niger Republic, Chad, Cameroon, and Republic of Benin [22], majority of eye care service practitioners are located in the cities [23] and in 2018, the country was home to about 4,000 registered optometrists [24] and 300 ophthalmologists [25] and this has increased over time. Ghana, with a population of about 31, 072, 945, is also located on the Gulf of Guinea, and shares borders with Togo, Burkina Faso, and Cote d’Ivoire [26]. The country operates the district health system which is regulated by the Ministry of Health at the national level whilst the Ghana Health Services [27], an independent public agency, provides public health services.

In both countries, the provision of eye care services starts at the district level, which usually consists of a team of ophthalmic nurses and optometrists with ophthalmologists present in a few facilities. The regional level consists of all three cadres of eye care professionals (ophthalmologists providing surgical services, optometrists providing clinical refraction while ophthalmic nurses are largely responsible for outpatient services). In hospitals where there are no ophthalmologists, ophthalmic nurses focus mainly on the medical management of ocular disease.

### Overview of optometry practice in Nigeria and Ghana

Optometrists in both Nigeria and Ghana are licensed to provide comprehensive primary eye-care services, such as refractive error correction, contact lens fitting, pediatric vision and binocular vision anomaly management, diagnosis and management of disease in the eye, and the rehabilitation of conditions of the visual system [20, 24, 28, 29]. Currently, only in both countries do optometrists in Africa complete a six year undergraduate professional Programme leading to the Doctor in Optometry degree [20, 28], which empowers them to provide general eye care including the glaucoma diagnosis and other eye diseases. Presently, there are two optometry schools in Ghana [30] and seven in Nigeria [31] all offering the OD degree. The two countries have similar curriculum for the OD program. Whereas Ghana commenced the OD program in 2002, the OD program has been running in Nigeria since 1981. The minimum qualification required to practice optometry in both countries is the OD degree. Furthermore, optometrists in both countries are required to be registered with their respective optometry regulatory agencies and the professional association as well as mandated to undertake continuing professional education to maintain their annual practice license. Majority of the practitioners are employed in private practices or own their practices usually in major cities which continue to accentuate the maldistribution of optometrists in both countries.

### Study population

At the time of the study, information from the respective professional associations revealed that there were 400 and 5271 licensed and practicing optometrists in Ghana and Nigeria, respectively. These included optometrists working in private and public service.

### Study design

This was a web-based cross-sectional survey of practicing optometrists in Ghana and Nigeria, conducted from 28^th^ March 2021 to 30^th^ June 2021. An e-link to the online survey designed on google form was emailed to the respective country’s optometry association for distribution to its members. The associations have databases containing the email addresses of all registered practitioners and all practicing optometrists in both countries are required to be registered with their respective associations. The associations also co-ordinate the activities of the profession. Both countries were selected because only in these countries are optometrist awarded a Doctor of Optometry degree and licensed to diagnose, treat, and manage glaucoma patients or diagnose, initiate treatment and refer patients with glaucoma. All optometrists who were registered with their respective local associations and had valid email addresses were informed about the study. Considering the low response rate in our previous study using similar distribution pattern [23], an e-link of the survey was also posted on optometry social media groups in both countries for a wider reach. However, to minimize repeated responses, participants were instructed in an online preamble not to complete the survey posted on social media if they had already done so.

### Sample size determination

The required sample size for this study was calculated differently for those in Ghana and Nigeria due to the difference in number of practicing optometrists in Nigeria (5271) and Ghana (400) using a single population proportion formula by World Health Organization [32]. The study assumed a proportion of 50% of the population (since no similar study has been carried out in Nigeria, or in Ghana) using a desired precision of 5% and 5% significance level for a two-sided test. This yielded a minimum required sample size of 385 for both countries which was considered adequate to detect any statistical difference in an analysis of online cross-sectional study to determine the diagnostic procedures and criteria for the diagnosis and management of glaucoma by practicing Nigerian and Ghanaian optometrists. To produce a country representative estimate of optometrists in the two countries, make comparison between countries even, and to further reduce bias from online surveys [33], the sample obtained from each country was weighted and the sampling weight was calculated as the reciprocal of the likelihood of being selected (inverse of the sampling fractions) in each country.

### Inclusion and exclusion criteria

All optometrists who, at the time of the survey, were involved in providing clinical services at different levels of eye care in Nigeria and Ghana and had at least one year working experience were eligible to participate. This was part of the preamble for completing the questionnaire. Participants were only able to progress to complete the survey after written informed consent had been obtained from them through responding with a ‘yes’ to the questions on whether they understood the study description and had voluntarily decided to take part in the study.

Exclusion criteria included ophthalmologists, non- Ghanaian optometrists practicing in Ghana, non-Nigerian optometrists practicing in Nigeria, Ghanaian and Nigerian optometrists practicing outside their countries of origin, optometrists working in non-governmental organizations who do not provide clinical services. We included optometrists in academic institutions since most institutions operate outpatient clinics where patient consultation is a part of the student clinical education.

### Survey tool

A self-administered questionnaire on the practice of glaucoma diagnosis among optometrists was used for data collection. The questionnaire was modified from similar studies [18, 34, 35] piloted and was approved by a panel of African optometrists and their responses were used to clarify questions that may not be clear to prospective respondents. Such questions were reworded and presented a second time before the final design was approved. Special instructions were provided on how to choose correct options necessary for ease of understanding. Additional file 1 (A1) shows the survey tool which contained largely multiple-choice questions based on the research objectives, with close-ended items and a few open-ended questions. The survey was designed in accordance with Checklist for Reporting Results of Internet Surveys (CHERRIES) guidelines [36].

The survey collected information on socio-demographic characteristics of the practitioners including age, gender, country of practice, location of practice, highest educational qualification, optometry training institution where they obtained their primary qualifications, years of practice experience; access to functional (essential) equipment for diagnosis and treatment of glaucoma; any glaucoma training completed post-graduation (duration of training, where training was completed); the process of diagnoses and management of glaucoma; whether they were aware of any preferred practice pattern for glaucoma diagnoses (e.g. International classification of diseases (ICD-10 or ICD-11) and what combination of glaucoma diagnostic tests they used for glaucoma diagnosis. Based on the recommended guidelines for glaucoma care [14–17], the combination of tests performed by the optometrists were then used to derive four levels of glaucoma assessment: Level 1 included optometrists who performed Intraocular pressure (IOP) measurement and optic nerve head examinations only; level 2 included optometrists who performed IOP measurement and optic nerve head and visual field examinations; level 3 (adequate care) included optometrists who performed all level 2 procedures and Optical Coherence Tomography (OCT) examinations; and level 4 (optimal care) included optometrists who perform all level 3 procedures and gonioscopy examination.

Questions were also asked about the availability of optometry equipment relevant to glaucoma practice including the type of OCT [37] used in their practices; estimated number of glaucoma patients per month, and type of glaucoma diagnosed in the last month. Finally, practitioners were asked to indicate the platform used in completing the survey. Further details on other questions are provided in the Additional file 1.

### Variables description

#### Dependent and independent variables

The dependent variable for this study was ‘practice of glaucoma care’, which was obtained from the question ‘do you diagnose glaucoma in your practice?’ with responses categorized as ‘1’ for ‘yes’ and ‘0’ for ‘no’. The independent variables were the demographic factors (age, gender, marital status, highest educational qualification, location of facility) and practice factors (type of service, mode of practice, ownership of practice, years of practice experience, and whether/not they attended any further training in glaucoma care and management. These were used to identify the factors associated with glaucoma practice in this study.

### Statistical analysis

Data collected online was exported from the Google Forms into Microsoft Excel spreadsheets (Microsoft Corporation, Redmond, WA, USA) and cross-checked for accuracy. The sampling weights for each country were used in the analyses to show the representativeness of this study. Data analysis was performed using Stata version 14.0 (Stata-Corp, College Station, TX, USA) with 'Svy' commands used to allow for adjustments of sampling weights used in the study when estimating confidence intervals around prevalence estimates, and the Chi-Squared test was used to test the strength of associations between demography and practice factors and glaucoma diagnosis.

‘Diagnosing glaucoma’ was treated as the dependent variable in the univariate, and multiple logistic regression analyses and were used to identify the factors associated with the diagnosis of glaucoma among optometrists in both countries. All variables with a statistical significance of *P* < 0.05 in the multiple logistic regression analyses were considered as the associated factors.

## Results

### Demographic characteristics of the participants with glaucoma

A total of 493 (more than the minimum required sample size of 385) optometrists from both countries responded to the survey; 238 (48.3%) and 255 (51.7%) from Ghana and Nigeria respectively. Due to the use of various platforms and the online distribution of the survey, response rate could not be calculated. Table 1 shows the distribution, demographic, and background information of respondents for the weighted and unweighted samples. There were more male optometrists in Ghana (61.7%), but slightly more female optometrists in Nigeria (52.3%). The age of practitioners from both countries ranged from 23 to 60 years, with a mean of 34.5 (SD 7.6) years. About two-thirds of the optometrists were practicing in urban areas (76.5%) with more than half involved in primary care services (55.4%). Predominantly, optometrists in this study worked in private practice (56.6%) particularly those in Nigeria (63.8%). In, Ghana there were more faith-based practices (facilities owned by religious groups) than Nigeria (16.7 vs. 2.0%). About one in every four optometrists (24.7%) were self-employed, suggesting that two-thirds 75.3% of those who were involved in private practice worked for others.

**Table 1:** Demographic and practice characteristics of Ghanaian and Nigerian optometrists in this study

Characteristics	Ghana		Nigeria	
	**(*n* = 238)**	**(*n* = 400)**^**a**^	**(*n* = 235)**	**(*n* = 5271)**^**a**^
**Demography**				
**Age in categories**				
< 35 years	168 (70.6)	282.4 (70.6)	124 (48.6)	2563 (48.6)
35–44 years	60 (25.2)	100.8 (25.2)	76 (29.8)	1571 (29.8)
45 + years	10 (4.2)	16.8 (4.2)	52 (20.4)	1075 (20.4)
** Gender**				
Male	183(76.9)	307.6 (76.9)	122(47.8)	2522 (47.8)
Female	55(23.1)	92.4 (23.1)	133(52.2)	2749 (52.2)
** Marital status**				
Married	126 (52.9)	211.8 (52.9)	248 (97.3)	3390 (64.3)
Not married	112 (47.1)	188.2 (47.1)	7 (2.7)	1860 (35.3)
** Location of facility**				
Peri-Urban	36 (15.1)	60.5 (15.1)	25 (9.8)	516.8 (9.8)
Rural	46 (19.3)	77.31 (19.3)	9 (3.5)	186 (3.5)
Urban	156 (65.6)	262.2 (65.5)	221 (86.7)	4568 (86.7)
** Highest qualification**				
Doctor of Optometry (OD)	211 (88.7)	354.6 (88.7)	205 (80.4)	4237 (80.4)
Master of Philosophy (MPhil)	20 (8.4)	33.6 (8.4)	22 (8.60	454.8 (8.6)
Doctor of Philosophy (PhD)	5 (2.1)	8 (2.1)	13 (5.1)	268.7 (5.1)
Bachelors (BSc)/others	2 (0.8)	1.681 (0.4)	12 (1.2)	62.0(1.2)
Fellowship	0 (0.0)	0 (0.0)	12(4.7)	248 (4.7)
**Practice factors**				
**Type of service**				
Tertiary	22 (9.2)	37 (9.2)	37 (14.5)	764.8 (14.5)
Secondary	47 (19.8)	79 (19.7)	42 (16.5)	868.2 (16.5)
Primary	107 (45.0)	179.8 (45.0)	78 (30.6)	1612 (30.6)
Not specified	62 (26.0)	104.2 (26.1)	98 (38.4)	2026 (38.4)
** Mode of practice**				
Government practice	111(46.6)	186.6 (46.6)	90(35.3)	1860 (35.3)
Private practice	125(52.5)	210.1 (52.5)	165(64.7)	3411 (64.7)
** Ownership of facility**				
Yes	39 (16.3)	65.5 (16.4)	82 (32.2)	1695 (32.2)
No	197 (82.7)	331.1 (82.8)	169 (66.3)	3493 (66.3)
** Practice years**				
< 2 years	38 (16.0)	63.9 (16.0)	37(14.5)	764.8 (14.5)
2—5 years	93 (39.1)	156.3 (39.1)	63 (24.7)	1302(24.7)
6–10 years	74 (31.1)	124.4 (31.1)	50 (19.6)	1034 (19.6)
11–20 years	33 (13.9)	55.46 (13.9)	63 (24.7)	1302 (24.7)
> 20 years	0 (0.0)		40 (15.7)	826.8 (15.7)
** Further training**				
No	107 (45.0)	179.8 (45.0)	132 (51.8)	2729 (51.8)
Yes	131 (55.0)	220.2 (55.0)	120 (47.1)	2480 (47.1)

^a^Weighting sample

In both countries, less than a quarter (14.9%) had achieved a higher qualification of Masters, Doctor of Philosophy, or Fellowship. About half of the respondents had between 1–5 years of practice experience. Respondents were asked to indicate if they had received any further training in glaucoma after their primary optometry qualification and their responses are presented in Table 1 including the proportion, duration, and the means through which they acquired the glaucoma care training. More than half (51.3%) reported receiving further training; slightly more Ghanaian optometrists (55.0%) had received training compared to the colleagues from Nigeria (47.8%) Table 1. Among those who had received further training in glaucoma care, the majority (76.2%) were through continuous education programs, two-thirds (67.9%) of which lasted less than a week.

### The practice of glaucoma care

Table 2 shows that significantly more Ghanaian optometrists reported that they diagnosed glaucoma compared with their Nigerian counterparts (98.7%; 95%CI:96.2–99.6 versus 93.7%; 95%CI: 89.9–96.1, *P* = 0.004). Of those who diagnose glaucoma, about 47.9% of Ghanaian and 45.6% of Nigerian optometrists diagnose less than 10 glaucoma patients per week and 39.3% and 46.8%, respectively, diagnose between 10–40 glaucoma cases weekly. The type of glaucoma cases commonly seen by practitioners varied significantly between both countries (*P* = 0.007), but primary open angle glaucoma was the single most diagnosed glaucoma type (Table 2).

**Table 2:** Prevalence (%) of glaucoma diagnosis, and test Procedures by country

Diagnosis of glaucoma	Ghana Prevalence (95% CI)	Nigeria Prevalence (95% CI)	*P–value*
**Diagnose glaucoma in practice (Yes)**	98.7 (96.2 – 99.6)	93.7 (89.9 – 96.1)	0.004
**Average no of cases seen per week**			
< 10	47.9 (41.5 – 54.3)	45.6 (39.3 – 52.0)	0.068
10–40	39.3 (33.2 – 45.7)	46.8 (40.5 – 53.2)	
41–70	10.3 (7.0 – 14.9)	4.6 (2.6 – 8.2)	
71–100	1.7 (0.6 – 4.5)	1.3 (0.4 –3.9)	
Over 100	0.9 (0.2 – 3.4)	1.7 (0.6 – 4.4)	
**Glaucoma types**			
POAG	32.5 (26.8 – 38.8)	27.8 (22.5 – 33.9)	0.007
POAG; CG	1.3 (0.4 – 3.9)	2.1 (0.9 – 5.0)	
POAG; PACG	5.1(2.9 – 8.8)	3.0 (1.4 – 6.1)	
POAG; PACG; CG	0.9 (0.2 – 3.4)	1.3 (0.4 – 3.9)	
POAG; PACG; SG	10.7 (7.3– 15.4)	19.0 (14.5 – 24.5)	
POAG; PACG; SG; CG	12.4 (8.7 – 17.3)	16.5 (12.2 – 21.8)	
POAG; SG	22.2 (17.3 – 28.0)	21.9 (17.1 – 27.7)	
POAG; SG, CG	14.5 (10.6 – 19.7)	8.4 (5.5 – 12.7)	
SG	0.0 (0.1 – 3.0)	^	
**Procedures performed – diagnose Glaucoma**			
Slit lamp examination of the anterior segment (Yes)	96.9 (93.7 – 98.5)	74.9 (67.9 – 80.8)	< .001
tonometry (Yes)	98.7 (96.1 – 99.6)	99.6 (97.0 – 99.9)	.316
Perimetry (Visual Field Testing) (Yes)	93.5 (89.4 – 96.2)	92.4 (87.9 – 95.3)	.638
*Fundus examination*			
Direct ophthalmoscope (Yes)	99.1 (96.6 – 99.8)	99.1 (96.6 – 99.8)	.993
Indirect ophthalmoscopy (Yes)^a^	31.9 (24.6 – 40.2)	41.1(32.2 – 50.7)	.136
Fundus photography (Yes)	34.7 (27.4 – 42.9)	57.4 (48.1 – 66.1)	< .001
OCT (Yes)	87.6 (82.3 – 91.4)	71.9(63.9 – 78.8)	< .001
Gonioscopy (Yes)	14.1 (9.0 – 21.3)	30.2 (21.8 – 40.2)	.003
*Pachymetry*			
Optical devices	64.2 (:53.1–73.9)	32.7 (24.0–42.6)	
Ultrasound	16.0 (9.5–25.8)	52.0 (42.1–61.8)	
**Practice standard**			
Level 1(IOP and ONH*)	2.1 (0.9 –5.0)	24.9 (19.9–30.7)	< .001
Level 2 (IOP, ONH and VFT)	16.9 (12.6–22.2)	34.1 (28.5–40.3)	
Level 3 (IOP, ONH and VFT and OCT)	75.5 (69.6–80.6)	29.7 (24.4–35.7)	
Level 4 (IOP, ONH and VFT, OCT and Gonioscopy)	5.5 (3.2–9.2)	11.2 (7.9 –15.8)	

*POAG* Primary open-angle glaucoma, *PACG* Primary angle-closure glaucoma, *SG* Secondary glaucoma, *CG* Congenital glaucoma, *IOP* Intraocular pressure, *ONH* Optic nerve head examination, *VFT* Visual field test, *OCT* Optical coherence tomography

^a^Includes biomicroscopy

**Fig. 1:**
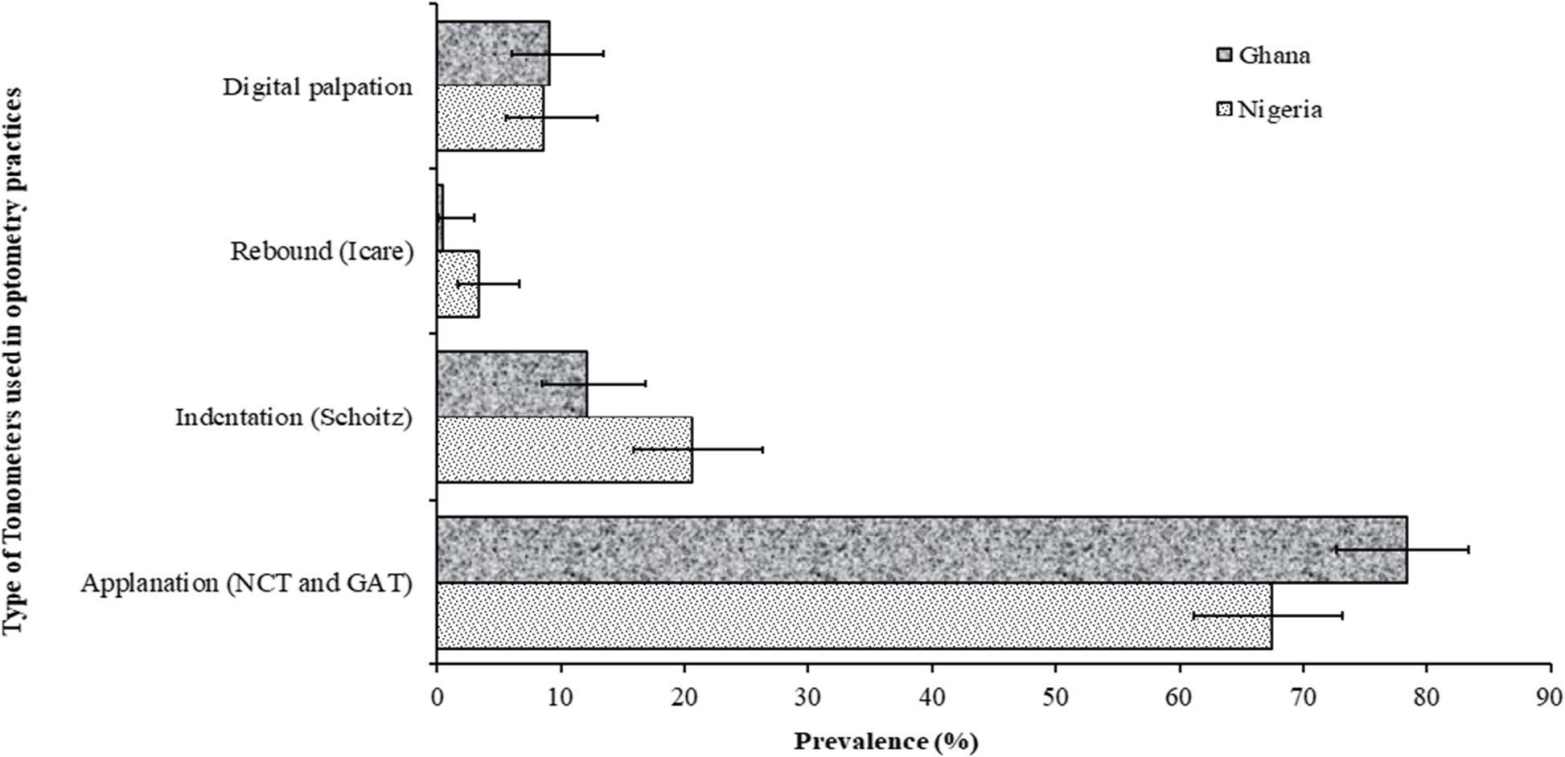
Prevalence and 95% confidence intervals of tonometer by types among Optometrist in Ghana and Nigeria. NCT: Noncontact tonometry, GAT (Goldman applanation tonometry)

**Fig. 2:**
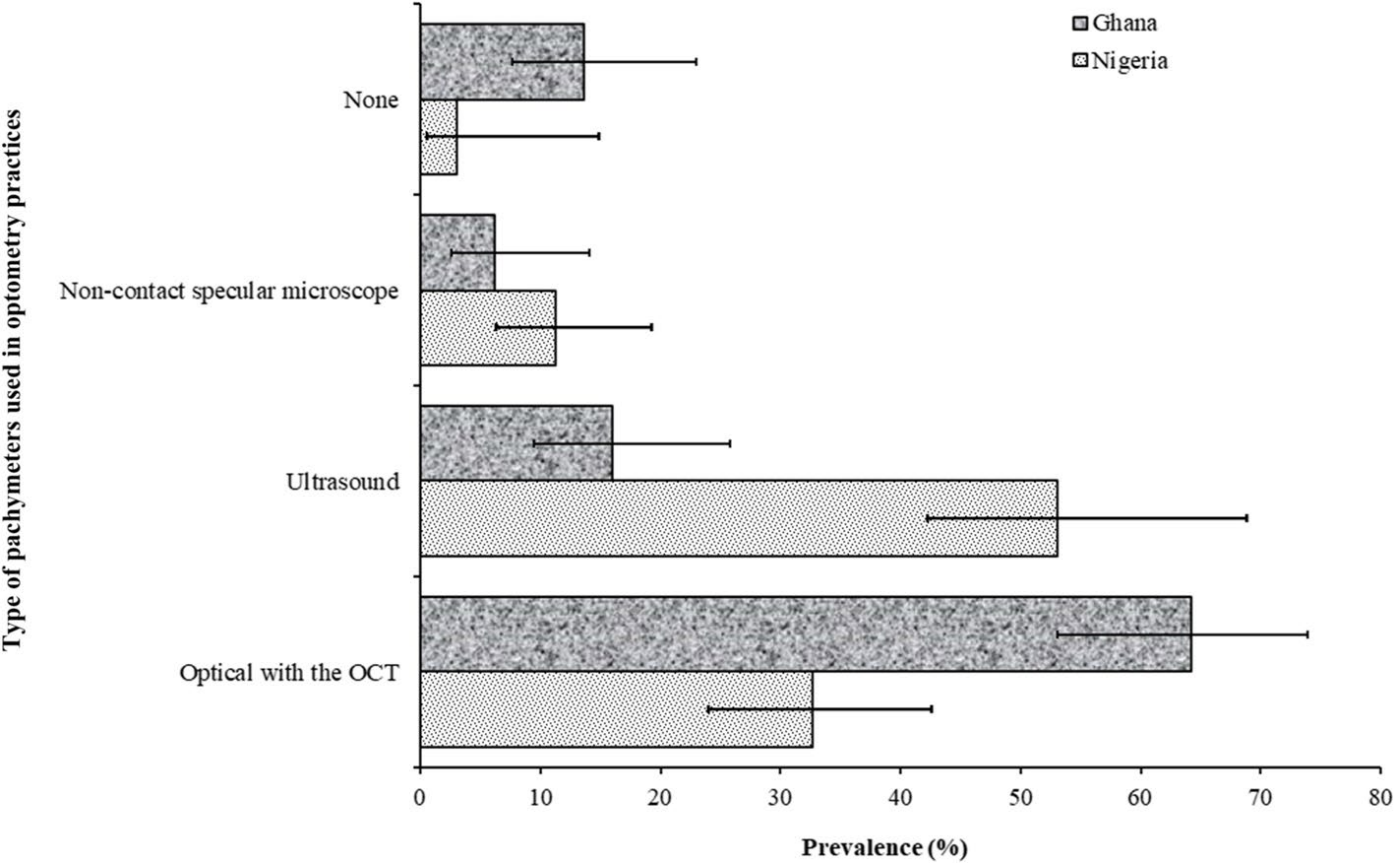
Prevalence and 95% confidence intervals of pachymeter by type among Optometrist in Ghana and Nigeria. OCT, Ocular coherence tomography

**Fig. 3:**
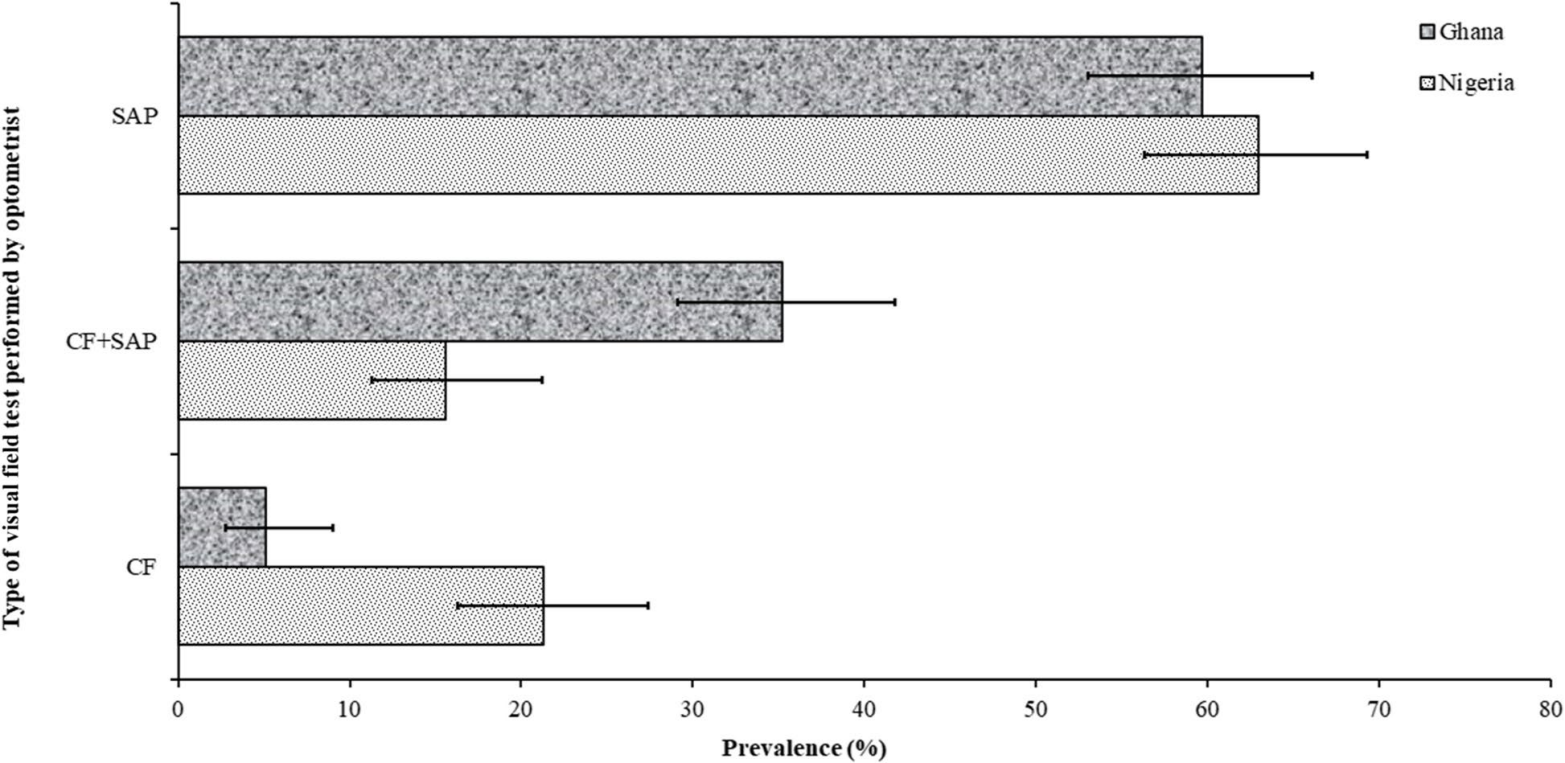
Prevalence and 95% confidence intervals of visual field techniques performed by optometrist in Ghana and Nigeria. SAP, standard automated perimetry; CF, confrontation visual field

Regarding the various procedures used to diagnose glaucoma by optometrists in both countries, it was observed that all the elements of the diagnostic procedures stipulated in guidelines by the American Academy of Ophthalmology, American Optometry Association, Australian National Health and Medical Research Council, and the UK’s National Institute for Health and Care Excellence were performed to varying extents in both countries. Tonometry, visual field testing, and fundus examination with direct ophthalmoscope were the prevalent techniques used by practitioners in both countries during diagnoses of glaucoma while gonioscopy was the least performed procedure. Comparison of the different techniques between countries is presented below (Figs. 1, 2 and 3).

### Tonometry

Although nearly all optometrists in both countries reported that they performed tonometry during glaucoma diagnoses, the preferred technique differed between countries (*P* = 0.01). Figure 1 shows the distribution of the tonometry techniques used by practitioners in both countries. Applanation tonometry was the most common technique used for measurement of IOP in both countries although predominantly used among Ghanaians (78.4%; 95%CI: 72.7–83.3 versus 67.4%; 95%CI: 61.1–73.1), who showed slightly lower preference for indentation tonometry than their Nigerian counterparts (12.1%; 95%CI: 8.5–16.9 versus 20.6; 95%CI: 15.9–26.3). Other techniques such as rebound tonometry (more among Ghanaians) and digital palpation were seldomly used by practitioners in both countries (Fig. 1). A breakdown of the applanation devices revealed that Noncontact tonometry was the preferred technique among Nigerian optometrists (28.9% versus 8.4%) while Goldmann applanation tonometry (GAT) was preferred among Ghanaian optometrists (40.8% versus 3.9%).

### Pachymetry

Measurement of corneal thickness during glaucoma diagnosis was routinely performed by 27.7% of Ghanaian and 39.8% of Nigerian optometrists (*P* = 0.009). Figure 2 shows the distribution of pachymetry techniques used by Optometrists in both countries. The proportion of Ghanaian optometrists who routinely performed pachymetry using optical devices almost doubled that in Nigeria (64.2%; 95%CI: 53.1–73.9 versus 32.7%; 95%CI: 24.0–42.6), whereas the proportion of Nigerian optometrists who routinely performed ultrasound pachymetry more than tripled those in Ghana (52.0%; 95%CI: 42.1–61.8 versus 16.0%; 95%CI: 9.5–25.8).

### Optic nerve assessment

Considering the techniques used for fundus examination, nearly all optometrists in both countries performed direct ophthalmoscopy, and the proportion who performed ocular coherence tomography examination during glaucoma assessment was slightly higher in Ghana than Nigeria (87.6%; 95%CI: 82.3–91.4 versus 71.9%; 95%CI: 63.9–78.8, *P* < 0.001). Nigerian optometrists reported significantly greater use of fundus camera for optic nerve assessment than the Ghanaian optometrists (57.4%; 95%CI: 48.1–66.1 versus 34.7%; 95%CI: 27.4–42.9, *P* < 0.001).

#### Gonioscopy

Although gonioscopy was seldomly used in both countries, its use was more among Nigerians (30.2%; 95%CI: 21.8–40.2 versus 14.1%; 95%CI: 9.0–21.3, *P* = 0.003).

### Visual field examination

The type of visual field test performed by optometrists in both countries is shown in Fig. 3. About 93% of practitioners in both countries reported that they routinely conducted a visual field test during glaucoma diagnosis and most performed standard automated perimetry (> 50%). A significantly higher proportion of Nigerian practitioners performed confrontational field test compared to the Ghanaians, who were more likely to routinely combine confrontational field with standard automated perimetry during assessment.

### Levels of glaucoma care

The categories of the four levels of glaucoma care are also shown in Table 2 and differed significantly between the two countries although most practitioners were practicing at the 2nd and 3rd levels of glaucoma care. More optometrists in Ghana were practicing at Level 3 standard of care while more Nigerians were practicing at levels 1 and 2. Very few Ghanaian optometrists (5.5%) and slightly more Nigerian optometrists (11.2%) were practicing at the level 4 which included performing all the recommended glaucoma diagnostic tests (Table 2).

### Association of demographic variables and practice of glaucoma care

Table 3 presents the prevalence of glaucoma care and the factors associated with practicing glaucoma care by optometrists in both countries. It shows a higher prevalence of glaucoma practice among Ghanaian optometrists (98.7%; 95%CI: 96.2–99.6) than Nigerians (93.7%; 95%CI: 89.9–96.1, *P* = 0.004) with a significant increase among practitioners who had more years of practice experience. The unadjusted analysis revealed that country of practice, years of practice experience and whether Optometrists received further training for glaucoma diagnosis were significantly associated with diagnosing glaucoma in Nigeria and Ghana. However, after adjusting for the potential confounders, Ghanaian optometrists (adjusted odd ratio, 5.86; 95%CI: 1.61–21.3) and those who have practiced for more than five years were significantly more likely to diagnose glaucoma compared with Nigerian optometrists and those who have practiced for 5 years or less.

**Table 3:** Prevalence and 95% confidence intervals (CI) of glaucoma diagnosis and factors associated with practice of glaucoma care among optometrist by country

Characteristics	Prevalence (95%CI)	OR (95%CI)	AOR (95%CI)	*P* value
**Demography**				
**Country**				
Nigeria	93.7 (89.9 – 96.1)**	1.00	1.00	
Ghana	98.7 (96.2 – 99.6)	5.29 (1.51 – 18.47)**	6.2 (1.6 – 23.2)**	.007
** Age in categories**				
< 35 years	91.1 (85.1 – 94.8)	1.00		
35–44 years	96.2 (89.0 – 98.8)	2.5 (0.7 – 9.1)		
45 + years	98.1 (87.7 – 99.7)	5.1 (0.6 – 40.0)		
** Gender**				
Male	93.3 (87.7 – 96.4)	1.00		
Female	94.8 (89.6 – 97.5)	1.32 (0.48 – 3.61)		
** Marital status**				
Married	95.9 (91.7 – 98.0)	1.00		
Not married	90.7 (83.2 – 95.0)	0.41 (0.15 – 1.13)		
** Location of facility**				
Peri-Urban	^	^		
Rural	93.0 (88.9 – 95.6)	^		
Urban	^	^		
** Highest qualification**				
Doctor of Optometry (OD)	93.1 (88.9 – 95.8)	^		
Master of Philosophy (MPhil)	^	^		
Doctor of Philosophy (PhD)	^	^		
Bachelors (BSc)/others	^	^		
Fellowship	^	^		
**Practice factors**				
**Type of service**				
Tertiary	86.6 (71.8 – 94.2)	1.00		
Secondary	97.8 (86.0 – 99.7)	7.0 (0.78 – 62.4)		
Primary	91.8 (83.9 – 96.1)	1.8 (0.52 – 5.85)		
Not specified	96.9 (91.3 – 99.0)	4.9 (1.17 – 20.7)*		
** Mode of practice**				
Government	88.6 (80.6 – 93.5)**	1.00	1.00	
Private	97.1 (93.3 – 98.7)	4.2 (1.46 – 12.3)*	3.3 (1.13 – 9.8)	.029
** Ownership of facility**				
Yes	97.6 (91.1 – 99.4)	1.00		
No	92.3 (87.5 – 95.4)	0.3 (0.07 – 1.3)		
** Practice years**				
< 2 years	81.9 (66.9 – 91.0)**	1.00	1.00	
2—5 years	94.1 (85.8 – 97.7)	3.5 (1.00 – 12.5)	2.19 (0.70 – 6.8)	.175
6–10 years	98.2 (88.3 – 99.8)	12.2(1.43 – 103.5)*	6.74 (0.68 – 67.3)*	.104
> 10 years	97.1 (91.5 – 99.1)	7.50 (1.8 – 30.6)**	7.04 (1.74 – 28.5)**	.006
** Further training**				
No	91.2 (85.2 – 94.9)	1.00		
Yes	96.9 (92.1 – 98.8)	3.07 (0.97 – 9.73)		

*OR* Odd ratio, *AOR* Adjusted odd ratio

^*^p < 0.05

^**^p < 0.01

## Discussion

This cross-sectional study investigated the practice of glaucoma care among optometrists in Ghana and Nigeria, two neighbouring countries with similar curriculum and graduating OD optometrists across Africa. The study revealed that although nearly all the optometrists from both countries reported that they diagnosed and co-managed glaucoma, there seems to be a lack of a developed preferred practice pattern for glaucoma. Such absence of a glaucoma practice guideline for optometrists prompted this study. We found that most optometrists performed the core elements of diagnostic testing which included tonometry, visual field testing, and fundus examination with direct ophthalmoscope, during glaucoma care. However, only a few (mostly Nigerian optometrists) reported that they often performed gonioscopy examination during glaucoma care. Very few optometrists in both countries (mostly Nigerian optometrists) were providing optimal care (level 4) for glaucoma. After adjusting for the potential confounders in the multivariate analysis, the study revealed that Ghanaian optometrists and those optometrists with more years of experience, were more likely to diagnose glaucoma compared with Nigerian optometrists and those with fewer years of experience, respectively. Similar findings were reported among optometrists in Australia and New Zealand, where the years of practice experience (10-15 years) was associated with improved glaucoma diagnostic practice [18].

As noted previously, optometrists in Nigeria and Ghana have the requisite training and legislation to diagnose and co-manage glaucoma [28]. The findings of this study suggested that streamlined national guidelines for the diagnosis and management of glaucoma are needed to ensure optimum evidence-based glaucoma care and to increase the likelihood of preserving patients’ vision and quality of life. Such national guideline should take into consideration the limited resources, remoteness and unavailability of equipment in these countries and should be incorporated into existing national eye health regulations. Despite the high prevalence of glaucoma practice among the optometrists, it is not exactly clear whether this can be extrapolated to all practicing optometrists in both countries. For the most part, many optometrists in this study practiced in urban areas representing a proportion of urban dwelling practitioners with access to the internet, relatively regular power supply, and modern ophthalmic instruments. This is supported by cogent evidence that over 60% of optometrists in Sub-Saharan Africa practice in urban and peri-urban areas leaving many rural areas underserved [38, 39]. Policies that encourage rural practice are needed in both countries.

The study found that broadly, optometrists in both countries performed the diagnostic procedures stipulated by guidelines of the American Optometry Association [15], American Academy of Ophthalmology [14], Australian National Health and Medical Research Council [17], and National Institute for Health and Care Excellence [16], albeit to varying degrees, but whether they knowingly perform the recommended procedures by these regulatory bodies remains unclear. It is more likely that optometrists in both countries perform these procedures without following the PPP guidelines but as a reflection of their training in optometry school. For example, about two-thirds of optometrists from both countries performed slit-lamp biomicroscope, which was comparable to the findings among optometrists in Australia (94.5% versus 98.9%) [40] given that majority of participants from both studies performed the procedure. Slit-lamp biomicroscope of the anterior segment is an important procedure that helps the clinician delineate glaucoma types and to rule out more aggressive forms of the disease like pseudo exfoliation and pigment dispersion glaucoma. However, while about 97% of Ghanaian optometrists performed slit lamp biomicroscopy, about 75% of Nigerian optometrists performed slit lamp biomicroscopy in the evaluation of glaucoma patients, which was still significantly less than that of Ghanaian optometrists even after adjusting for years of experience. It is not exactly clear why this difference exists since optometrists in both countries have same qualifications, but this could probably be priority during testing. Access and ease of importation of ophthalmic materials may explain the difference in ophthalmic practice but, understanding those factors were beyond the scope of this study and may need further research.

IOP remains one of the most important risk and modifiable factors [41] with GAT being the gold standard technique for measuring IOP [14–17]. In this study, majority of optometrists (97.5%) performed tonometry using different techniques, with non-contact tonometer and GAT, being the most frequently used instruments. Among Ghanaians, GAT was the most frequently used tonometer followed by noncontact tonometry (40.8% vs 8.4%) whereas in Nigeria, it was noncontact tonometer (28.9%) followed by GAT (3.9%). The preference for a particular form of tonometry may reflect individual access to the equipment rather than difference in training of optometrists in the two countries. Essentially, optometry institutions in both countries use the same curriculum for training and therefore the variation in glaucoma practice pattern may not be attributed to the optometry training curriculum in both countries. An important point to note here is that there are different types and designs of noncontact tonometer, whereas the GAT is a single standardized device. By comparison, 88.3% of Australian and 93.8% of New Zealand optometrists [40] perform GAT. It may be tempting to assume that access to GAT stemming from differences in the economies of these countries may explain the difference in the proportion of optometrists who perform GAT in Ghana and Nigeria compared to Australia and New Zealand [18]. Perhaps other factors such as the relative cost (compared to GAT which requires slip lamp biomicroscopy) and ease of performing noncontact, increased reliability and repeatability of new noncontact tonometer, and a lack of knowledge of the preferred practice guideline for tonometry in glaucoma care may account for the wide disparities.

The ocular hypertension treatment study demonstrated that subjects with thin central cornea thickness (CCT) were more likely to become glaucoma sufferers, making this parameter (CCT) an independent risk factor for glaucoma development [42]. Since that landmark study, the evaluation of CCT has become part of the preferred practice pattern recommended by the various international bodies. The proportion of optometrists in Ghana and Nigeria who routinely measured CCT using pachymetry on glaucoma patients were 27.7% and 39.8% respectively. In contrast, a higher proportion of optometrists in Australia (51.2%), a similar proportion in New Zealand (36.8%) [18], and a lower proportion in the UK (7%) [43] performed CCT measurements. These represent a relatively low proportion of optometrists who performed CCT measurement across the aforementioned countries but may increase in Australia and New Zealand, where the practice scope of optometrists was increased in 2014 and so it is likely that more optometrists now may be diagnosing and managing ocular diseases. In retail-based eye clinics in the United States, Stanley et al. [44] found a compliance to the American Academy of Optometry glaucoma practice guideline of 88.6% among optometrists. The fact that most U.S states have wider scope of practice and more robust optometry legislation that allow optometrist full range of practice may explain the outlier status of U.S optometrists.

The importance of anterior chamber angle assessment in glaucoma has been highlighted in the literature [45–47]. The optimum glaucoma management strategy depends on the state of the anterior chamber angle. Whether the angles are closed, open, have evidence of inflammation, neovascularization, pigment dispersion, or pseudo-exfoliation, will influence how glaucoma is managed. The gold standard and recommended technique for anterior chamber angle evaluation is gonioscopy, however, this was rarely performed by optometrists in both countries, especially those in Ghana. Similar lower proportions have been reported among optometrists in Australian and New Zealand in one study [18], and in the UK [43], with more optometrists in the United States of America performing gonioscopy (45%) [43, 44] than our study. Another study reported higher proportions among Australian (77.8%) and New Zealand (94.6%) optometrists [40]. The fact that a low percentage of practitioners performed this relatively inexpensive technique may be attributed to the steep learning curve required to master the procedure.

Visual field examination is used to establish the functional integrity of the optic nerve and is integral to diagnosing and monitoring glaucoma progression. The gold standard technique recommended in major professional guidelines is the standard automated perimetry. Although the full threshold standard automated perimetry strategy is better at eliciting early localized defects, it takes long to complete, leading to patient fatigue and reduction in the reliability indices of the test. This has spurred the development of algorithms that optimizes efficient detection of defects while minimizing test time. Examples of such algorithms include the Humphrey’s Swedish interactive threshold algorithm standard (SITA-standard) and the Octopus’ tendency-oriented perimetry [48, 49]. Compared with the optometrists in Australia (81%) and New Zealand (76%), a significantly lower proportion of those in Ghana and Nigeria (a little more than half) performed the standard automated perimetry technique solely and this could be explained by differences in economic access between the countries. A used Humphrey’s visual field analyzer could cost almost $5,000 to procure (precision equipment) which could be prohibitive to many optometry practices in sub–Saharan Africa.

A generalized or focal notch of the neuro-retinal rim tissue including a defect of surrounding retinal nerve fiber layers is pathognomonic of glaucomatous optic neuropathy [50]. A careful assessment and documentation of the optic nerve head and surrounding tissues are therefore important to rule out glaucomatous optic neuropathy. Recommended techniques for assessing the optic nerve head include direct and indirect ophthalmoscopy, and fundus photographic imaging. Most Ghanaian and Nigerian optometrists evaluated the optic nerve head with direct ophthalmoscopy, or with indirect ophthalmoscopy and about 57.4% and 34.7% of Ghanaian and Nigerian optometrists, respectively documented the health of the optic nerve head with fundus photography. On the other hand, only 21.1% of optometrists in Australia and New Zealand documented the health of the optic nerve head with fundus photography. The proportion of optometrists in this study who assessed the optic nerve head with the recommended standard instruments was similar to those in the developed economies which may suggest that optometrists in Ghana and Nigeria have the tools needed for the adequate examination of the optic nerve head in glaucoma care.

The OCT has become one of the central procedures in evaluating the structural integrity of the optic nerve and macula in glaucoma patients. Since its introduction in 1991 [51], this technology has made it easy to quantify the retinal nerve fiber layer thickness, the ganglion cell complex thickness, the neuro-retinal rim tissue area, the cup volume and more. Although the instrument is relatively expensive, we found that compared to reports among Australian and New Zealand optometrists [18], more optometrists in this study utilized this technique for the diagnosis of glaucoma. The significant number of Ghanaian and Nigerian optometrists who have access to and utilize the OCT technology is encouraging and implies that these optometrists have the diagnostic tools to detect glaucoma at the early stages of the disease.

Another finding of this study was that Ghanaian optometrists were more likely to diagnose glaucoma than their counterparts in Nigeria, which could be attributed to other factors such as funding for glaucoma care which although suboptimal in the region [52] exists in Ghana [53] but not in Nigeria [54], easier access to importation of ophthalmic equipment in Ghana, and more access to loan financing, which were not covered in this study. Additional analysis from this dataset also revealed that at the time of this study, more Ghanaian optometrists worked in government practices compared to the Nigerian optometrists (24 vs. 16%) who predominantly worked in private practice (36 vs. 26%). In this region, primary glaucoma care is more likely to take place in government hospitals where ophthalmologists primarily provide glaucoma care. Therefore, optometrists who work in the government hospitals are more likely to adhere and practice with a preferred pattern of care. However, further studies are needed to investigate the role of these factors in glaucoma practice across Africa.

### Limitations and strengths

The study has some limitations. First, it is possible that only the optometrists with an interest in glaucoma practice may have responded to the questionnaire, which might lead to overestimation or underestimation of our findings. Second, the survey responses are also susceptible to self-selection bias because respondents with access to new ophthalmic instruments are more likely to be confident in their clinical abilities and therefore more likely to respond to surveys. Third, there is the limitation associated with social-desirability bias where respondents may engage in a purposeful image impression management to fit into desirable expectation [55]. It is therefore possible that the actual practice pattern of the respondents in their clinics may not correspond with their responses as noted previously [56], but the non-availability of any objective data on patients' care or utilization data makes it difficult to confirm the subjective responses in this study. Fourth, we did not access their competence in interpretation of the tests such that Optometrist could be doing the imaging studies like OCT and VF but may not be competent in interpreting the same. Fifth, the responses of the optometrists regarding equiment use and general care of glaucoma in this study may be impacted by the fact that some do not have access to regular supply of electricity which most of the equipment need to operate. Sixth, another limitation of this study relates to the low response rate from Nigeria relative to the number of optometrists in comparison to Ghana, which has been noted in other studies (cite the ECP paper). However, unlike the past studies, we applied sample weighting to make our findings more representative of the optometry population sample in both countries. Considering the limitations, the results of this survey should be interpreted with caution and future studies are needed to provide evidence in the African region, which can then be used to either confirm or support our findings. Despite these limitations, this study has numerous strengths in addition to the sample weighting. The study provides valuable insights into the knowledge and awareness of optometrists on standard of care for glaucoma diagnosis. The study identified several gaps in the knowledge, awareness, and practice of glaucoma care among optometrists in the two sub–Saharan African countries with Doctor of Optometry degree. The findings will help in developing evidence-based resources which can be used as guides to create regional specific resources or refine existing guidelines for optometrists such as clinical practice guidelines and continuing professional education.

## Conclusions

This study indicated that although more than 90% of optometrists in both countries diagnose glaucoma, Ghanaian optometrists were more likely to do so, especially those who work in private practice and the more experienced practitioners. Majority of the optometrists are well-trained to diagnose glaucoma and are practicing at a reasonably adequate level for glaucoma care with some improvement in gonioscopy practice. The gaps identified in this study can be improved with better equipment resourcing to the optometrists and more frequent continuing education and training, with specific emphases on preferred practice guidelines. The findings also suggest that economic constraints may affect access to more capital-intensive diagnostic instrumentations, especially among Nigerians and public practice optometrists.

### Supplementary Information

**Additional file 1: Supplementary file S1.** Sample of the survey tool. **Supplementary file S2.** Consortium name and members.

## Data Availability

The datasets used and/or analysed during the current study are available from the corresponding author on reasonable request.

## Abbreviations

AOR: Adjusted odds ratio
CCT: Central corneal thickness
CI: Confidence interval
GAT: Goldmann applanation tonometer
IOP: Intraocular pressure
OCT: Optical coherence tomography
OD: Doctor of optometry
OR: Odds ratio
POAG: Primary open angle glaucoma
SITA: Swedish interactive threshold algorithm
WHO: World Health Organization
