# Supplementary material for: Profiling and factors associated with glaucoma diagnostic practice in sub-Saharan Africa-a cross sectional study of Nigerian and Ghanaian optometrists
Source: BMC Ophthalmol. 2023 Aug 8;23:351. doi: 10.1186/s12886-023-03083-0 (PMC10410918; doi:10.1186/s12886-023-03083-0)
Supplement: Supplementary file 1 — Additional file 1: Supplementary file S1. Sample of the survey tool. Supplementary file S2. Consortium name and members. [file 12886_2023_3083_MOESM1_ESM.docx]

**Supplementary file S1: Sample of the survey tool**

**Section A: Socio-demographic characteristics and practitioners’ background**

1. Are you an optometrist Yes/No
2. What is your gender:
   - Male
   - Female
   - Others
3. Select your country of practice?

- Ghana
- Nigeria

Please specify City/town………………..

1. Where is your practice located?
   1. Rural
   2. Urban
   3. Peri-urban

4b. If you practice in Government facility, which level of care is your practice

- Primary
- Secondary
- Tertiary

1. Age: __________________
2. Marital Status:

- Single
- Married
- Separated/divorced
- Widow/widower

1. What is your highest qualification..
   - BSc
   - OD
   - Masters
   - PhD
   - Other (including fellowship)
2. How long have you been in practice? (Pick the range that applies)
   - < 2 years
   - 2 – 5 years
   - 6 – 10 years
   - 11 – 20 years
   - > 20 years
3. In which Optometry school did you obtain your primary training?

- Abia State University, Okigwe (ABSU: includes those who studied at Etiti)
- Federal University of Technology Owerri (FUTO)
- Imo State University Owerri (IMSU, excluding those who studied at Etiti)
- MADONNA
- University of Benin (UNIBEN)
- University of Cape Coast
- Kwame Nkrumah University of Science and Technology
- Others (Specify)

1. Have you had a further training in glaucoma care since graduation? Y/N

- Yes skip to question 11
- No Skip to question 13

1. What was the duration, please specify in days, weeks, months and years. For e.g, 6 days

………….

1. Where were you trained. Please specify the institution or organization/school………...

**Mode of practice**

1. Which of these describes your mode of practice?

- private optometry practice including own practice
- private ophthalmology practice
- Group private practice with both ophthalmologists and optometrists.
- Government hospital (includes university clinic/teaching hospitals, military hospitals).
- Quasi-government facility
- Others (specify)

1. Do you own your practice? Yes/No

**Section B: Glaucoma Practice standard** **and diagnostic criteria**

1. Do you diagnose glaucoma in your practice? (Y/N)
2. If yes, which of these procedures/tests do you perform routinely during glaucoma evaluation? (Tick all that apply)

- Slit lamp examination of the anterior segment
- Tonometry
- Perimetry (Visual Field Testing)
- Fundus assessment with direct ophthalmoscope
- Fundus assessment with biomicroscopy
- Fundus imaging with fundus camera
- Fundus imaging with OCT
- Gonioscopy
- Others: specify

1. Which tonometer do you use in diagnosing glaucoma? (pick all that apply)

- Digital by palpation
- Schiotz
- Tonopen
- Handheld Perkins
- Slit lamp Mounted Goldman Applanation
- Air-puff Non-contact
- Dynamic contour
- Ocular response Analyzer
- Other: specify

1. Is corneal thickness assessment with a pachymeter part of your routine evaluation of glaucoma? (Y/N) (***If No, please proceed to Question 20)***
2. If yes, what type of pachymeter do you presently use? Choose the option(s) that apply to you

- Ultrasound
- Non-contact specular microscope
- Ocular coherence tomography
- Others: Specify………………

1. Do you evaluate the optic nerve in your glaucoma assessment? (Y/N) ***If No, please proceed to Question 22)***
2. If yes, how do you evaluate the optic nerve? (Pick all that apply)

- Direct Ophthalmoscopy
- Indirect Ophthalmoscopy including BIO
- Fundus photography
- OCT
- Other: specify

1. If you perform visual field examination for glaucoma assessment, what type of visual field technique do you use? Pick all that apply
   1. Confrontational visual field
   2. Tangent screen
   3. Manual perimetry
   4. Standard Automated Perimetry (SAP)
2. Do you perform gonioscopy during evaluation of a glaucoma patient? (Y/N)
3. Do you use the Optical coherence tomography (OCT) instrument in your place of practice for glaucoma diagnosis? (Y/N)

**Questions on glaucoma patients**

1. On the average, how many glaucoma diagnoses did you make in the past month, in your practice?

- Less than 10
- 11-40
- 41-70
- 71-100
- Over 100

1. What type of glaucoma have you diagnosed in your practice? (Pick all that apply)

- Primary open angle glaucoma
- Primary angle closure glaucoma
- Secondary glaucoma
- Congenital glaucoma

**Section D: Question on Pattern of Glaucoma diagnosis**

1. Which combination of glaucoma diagnostic tests do you use in the diagnosis of glaucoma (pick one option that most apply to you)

- Tonometry and optic nerve evaluation
- Tonometry, optic nerve evaluation, and VFT (threshold SAP)
- Tonometry, optic nerve evaluation, VFT, and OCT
- Tonometry, Gonioscopy, optic nerve evaluation, VFT, and OCT

Thank you for participating

**Supplementary file S2: Consortium name and members**

**Centre for Eye Care and Public Health Intervention Initiative (CEPHII) Members**

Stephen Ocansey^1ǂ^, Edgar Ekure^2ǂ^, Uchechukwu L. Osuagwu^3,9^*, Bernadine N. Ekpenyong^4^, Godwin Ovenseri-Ogbomo^5^, Sylvester Kyeremeh^6^, Kelechi C. Ogbuehi^7^, Kingsley E. Agho^8,9^ , Khathutshelo P. Mashige^9^, Jyoti Naidoo^9^, Antor O. Ndep^10^, Kovin S. Naidoo^9,11^ , Tuwani Rasengane^12^,

**Affiliations**

^1^Department of Optometry and Vision Science, School of Allied Health Sciences, College of Health and Allied Sciences, University of Cape Coast, Ghana.

^2^Department of Biomedicine, Salus University, 8360 Old York Road, Elkins Park, PA 19027, USA.

^3^Bathurst Rural Clinical School (BRCS), Western Sydney University, New South Wales 2795, Australia.

^4^Epidemiology and Biostatistics Unit, Department of Public Health, University of Calabar, Cross River State, Nigeria.

^5^Department of Optometry, Centre for Health Sciences, University of the Highlands and Islands, Inverness, IV2 3JH, UK.

^6^Department of Optometry and Visual Science, College of Science, KNUST

^7^Department of Medicine, Dunedin School of Medicine, University of Otago, New Zealand.

^8^School of Health Science, Western Sydney University, Campbelltown, NSW 2560, Australia.

^9^African Vision Research Institute, Discipline of Optometry, University of KwaZulu-Natal, Westville Campus, Durban, 3629, South Africa.

^10^Department of Public Health, Faculty of Allied Medical Sciences, College of Medical Sciences, University of Calabar, Cross River State, Nigeria.

^11^School of Optometry and Vision Science, University of New South Wales, Sydney, New South Wales, Australia.

^12^Department of Optometry, University of the Free State and Universitas Hospital, Bloemfontein, South Africa
